# Role of Pyridine Nitrogen in Palladium-Catalyzed Imine Hydrolysis: A Case Study of (E)-1-(3-bromothiophen-2-yl)-N-(4-methylpyridin-2-yl)methanimine

**DOI:** 10.3390/molecules24142609

**Published:** 2019-07-17

**Authors:** Gulraiz Ahmad, Nasir Rasool, Komal Rizwan, Ataf Ali Altaf, Umer Rashid, Mohd Zobir Hussein, Tariq Mahmood, Khurshid Ayub

**Affiliations:** 1 Department of Chemistry, Government College University, Faisalabad 38000, Pakistan; 2 Department of Chemistry, Government College Women University, Faisalabad 38000, Pakistan; 3 Department of Chemistry, University of Gujrat, Hafiz Hayat Campus, Gujrat 50700, Pakistan; 4 Institute of Advanced Technology, University Putra Malaysia, 43400 UPM Serdang, Selangor, Malaysia; 5 Department of Chemistry, COMSATS University Islamabad, Abbottabad Campus, University Road, Tobe Camp, Abbottabad 22060, Pakistan

**Keywords:** Thiophene, carbaldehyde, hydrolysis, cleavage, palladium, imine.

## Abstract

In the present study, 4-methylpyridin-2-amine was reacted with 3-bromothiophene-2-carbaldehyde and the Schiff base (E)-1-(3-bromothiophen-2-yl)-N-(4-methylpyridin-2-yl)methanimine was obtained in a 79% yield. Coupling of the Schiff base with aryl/het-aryl boronic acids under Suzuki coupling reaction conditions, using Pd(PPh_3_)_4_ as catalyst, yielded products with the hydrolysis of the imine linkages (**5a**–**5k**, **6a**–**6h**) in good to moderate yields. To gain mechanistic insight into the transition metal-catalyzed hydrolysis of the compounds, density functional theory (DFT) calculations were performed. The theoretical calculations strongly supported the experiment and provided an insight into the transition metal-catalyzed hydrolysis of imines.

## 1. Introduction

Imines are key intermediates for the synthesis of many bioactive compounds [1]. Imines can be easily synthesized and form complexes with various metal ions in different oxidation states. A number of imine derivatives have been reported for versatile pharmaceutical activities including analgesic [2], antimicrobial [3], antioxidant [4], anticancer [5], anticonvulsant [6], and antihelmintic [7]. Imines are well known for the development of inorganic biochemistry and coordination chemistry, as well as applications in a variety of chemical areas such as oxidation, reduction, and hydrolysis catalysis [8]. Hydrolytic cleavage of the C=N occurs on a number of metal sites ranging from simple salts to mixed ligand complexes [9,10]. The reaction conditions, co-ligands and solvent systems are also main factors [11,12,13]. Gourbatsis et al. [14] reported the role of metal ions in the hydrolysis of imines. According to various reports [11,15,16,17] the hydrolysis of imines is dependent on different factors: The pH of the medium, the size of the chelate rings, the coordinating ability of the counter anions, the nature of the metal ions, and the effect of carbonyl compounds.

This project was designed for synthesis of novel pyridine-based imines via the Suzuki coupling reaction and density functional theory (DFT) calculations were performed for studying the mechanistic pathway of hydrolysis, which was possibly a side reaction during the synthesis.

## 2. Results and Discussion

### 2.1. Chemistry

**Scheme 1:**
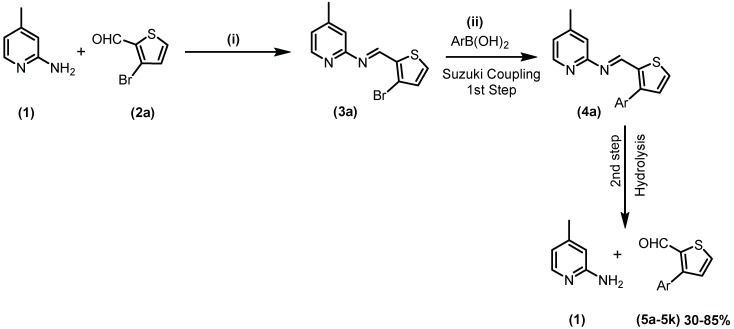
Synthesis and Suzuki coupling of (E)-N-((3-bromothiophen-2-yl)methylene)-4-methylpyridin-2-amine Schiff base (**3a**) with arylboronic acids.

In this research, commercially available 4-methylpyridin-2-amine (**1**) was reacted with 3-bromothiophene-2-carbaldehyde (**2a**) in the presence of glacial acetic acid and the Schiff base (E)-1-(3-bromothiophen-2-yl)-N-(4-methylpyridin-2-yl)methanimine (**3a**) was obtained in a 79% yield. The heterocyclic Schiff base (**3a**) containing halide was coupled with aryl/het-aryl boronic acids under Suzuki coupling reaction conditions, using Pd(PPh_3_)_4_ as the catalyst. However, the reactions did not stop at the first step stage, illustrated in Scheme 1, and we were unable to get the expected products. We attempted a number of experiments with variations of boronic acids and every time we got products with the hydrolysis of the imine linkage, as shown in the second step of Scheme 1. The products (**5a**–**5k**) after hydrolysis were obtained in 30–85% (Scheme 1, Table 1).

In the literature, a number of examples are reported by different research groups for such a kind of imine hydrolysis on the metal center during the formation of the coordination complex. Such a kind of imine hydrolysis depends on various factors, such as the pH [17], the size of the chelate rings [18], counter anions [19,20], the nature of the metal ions [18], and the effect of carbonyl compounds [21]. Despite our thorough literature research, we were unable to find any such examples, which lead to catalytic reactions of amine hydrolysis with metal (i.e., the formation of free hydrolysis products). In the current study, a Pd-complex acts as a catalyst for the imine hydrolysis reactions.

**Scheme 2:**
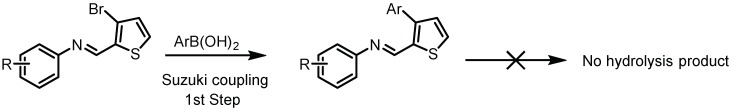
Previous report of Suzuki coupling of a Schiff base by our group [22].

To understand the pathway of imine hydrolysis in these reactions, we repeated the experiments with variations of the reaction conditions. In the 1st set, the experiment was performed in the absence of a Pd catalyst, which resulted in non-hydrolysis of the imine linkage and obviously no Suzuki coupling. In the 2nd set, the experiment was performed without boronic acid derivatives (i.e., the reaction between the Schiff base-containing halide and the Pd catalysts). This experiment yielded the imine hydrolysis product. In the 3rd set, a Schiff base without a pyridine group was utilized for the Suzuki coupling, which yielded a non-hydrolysis product, as shown in Scheme 2 and reported by our group [22].

Such an observation may lead someone to think that there may exist some interaction between the pyridine nitrogen and the Pd atom of the catalyst, which leads to the decrease in the electronic charge density on the imine (C=N) group and ultimately the hydrolysis.

**Scheme 3:**
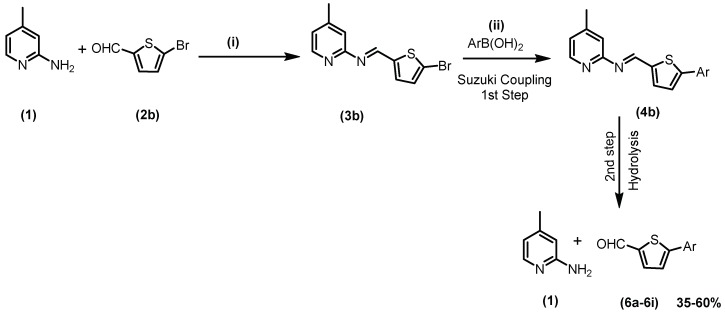
Synthesis and Suzuki coupling of (E)-N-((5-bromothiophen-2-yl)methylene)-4-methylpyridin-2-amine (**3b**) with aryl/het-aryl boronic acids.

**Scheme 4:**
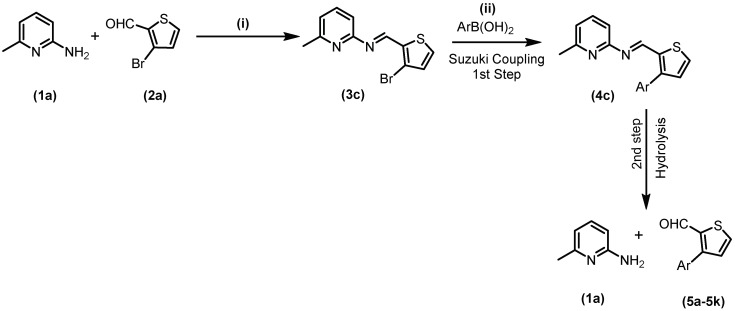
Synthesis and Suzuki coupling of (E)-N-((3-bromothiophen-2-yl)methylene)-6-methylpyridin-2-amine (**3c**) with aryl/het-aryl boronic acid.

**Scheme 5:**
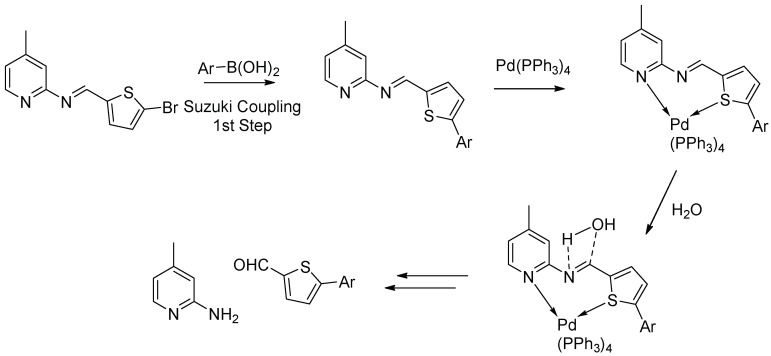
Plausible mechanism for the imine hydrolysis. The association of the Suzuki coupling product with the Pd-catalyst is due to the presence of the pyridine ring, this attachment increases the polarity of the C=N linkage of the Schiff base and ultimately leads to hydrolysis products. The absence of the pyridine ring does not allow such attachment.

To strengthen the idea, the reaction was performed by changing the position of the halogen, as shown in Scheme 3, which yielded similar hydrolysis products (**6a**–**6i**, 35–60%) (Table 2). A set of reactions was also performed by changing the position of the methyl group on the pyridine, as shown in Scheme 4, which yielded the same hydrolysis products (**5a**–**5k**). However, pyridine has some substantial role in the hydrolysis of the imine group. From these observations, the plausible mechanism for the imine hydrolysis is illustrated in Scheme B. The first step of the Suzuki reaction is oxidative addition, which leads to the formation of the Pd(II) complex (indicated by reaction’s first step product in Scheme 5. As a result of the oxidative addition, Pd becomes electrophilic and attracts electronic charge density from the carbon attached to it, which results in the decrease of electronic charge density at the imine (C=N) group. On the other side, the pyridine nitrogen also attracts an electron by the inductive effect and makes the C=N group more electron deficient as compared to the C=N group in the Schiff base without the pyridine group (Scheme 2). This more electrophilic C=N group allows the nucleophilic oxygen of water to attack for the hydrolysis, as shown in Scheme 5.

### 2.2. Mechanistic Studies via DFT Calculations for the Hydrolysis of Imine

**Figure 1:**
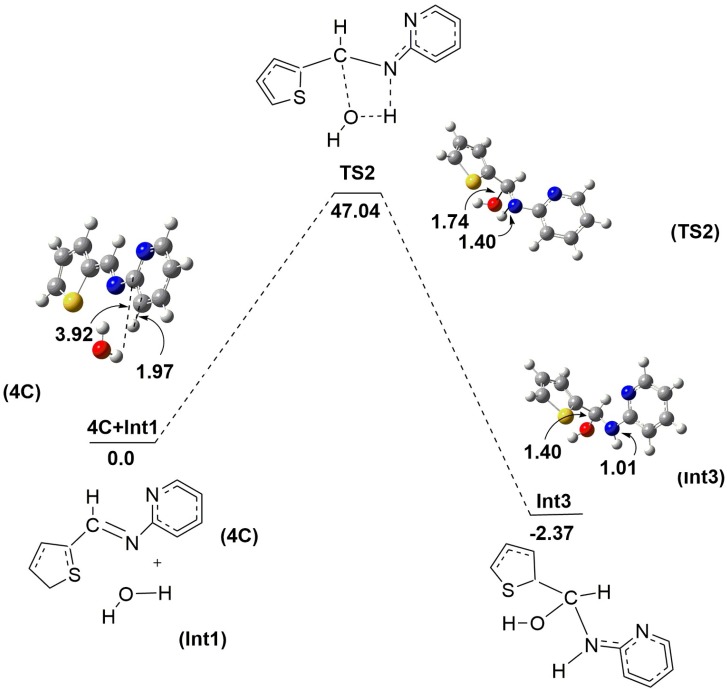
Hydrolysis pathway without the involvement of Palladium as a catalyst.

Density functional theory calculations (DFT) were performed to gain mechanistic insight into the transition metal-catalyzed hydrolysis of compound 4c. For this purpose, hydrolysis in the presence of a transition metal is compared with the reaction in the absence of transition metals. Moreover, for transition metal-assisted hydrolysis, the role of the pyridine moiety is also evaluated. The hydrolysis is believed to take place after the Suzuki coupling. Therefore, the product of the Suzuki coupling was taken for the DFT calculations. However, in order to reduce the computational cost, the Ar moiety (incorporated through the Suzuki coupling) was omitted during calculations. For metal free hydrolysis, a van der Waals type complex was observed as the starting species (Int1 and 4C in Figure 1). In this complex, a hydrogen of the water molecule interacts with the imine nitrogen through hydrogen bonding (1.97 Å). A transition state for hydration was located at a barrier of 47.04 kcal mol^−1^. The transition state clearly showed the formation of C-O and N-H bonds with concomitant cleavage of the O-H bonds. The C-O, N-H and O-H bond lengths in the transition state were 1.74 Å, 1.40 Å and 0.97 Å, respectively. High kinetic demand for this reaction clearly illustrates that the reaction was not feasible under reaction conditions. Although kinetically unfavorable, the reaction was thermodynamically favorable by 2.37 kcal mol^−1^.

**Figure 2:**
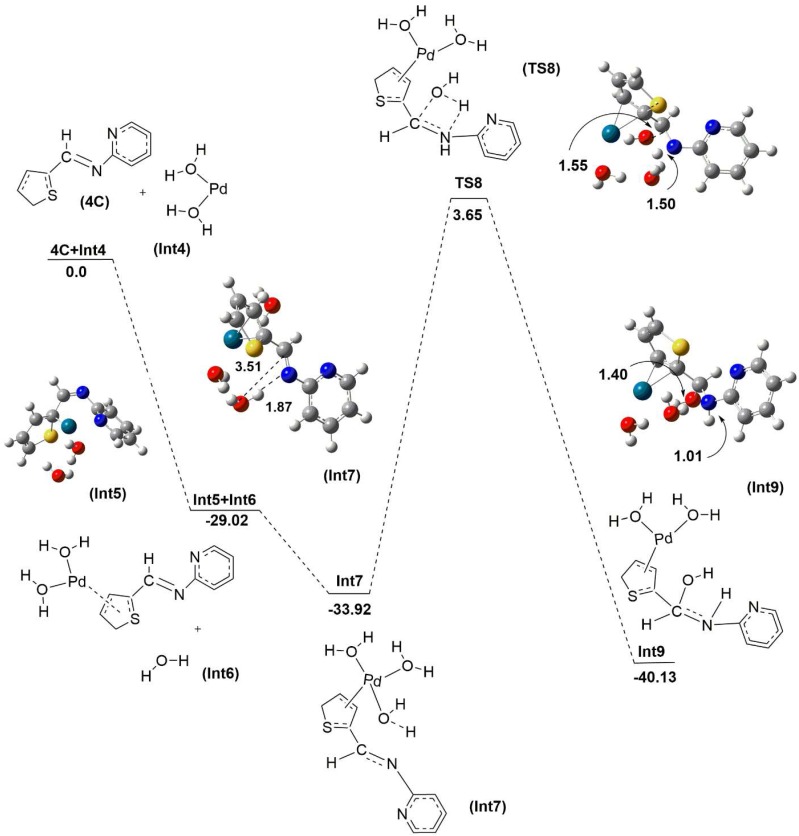
Hydrolysis pathway in the presence of palladium but without involvement of pyridine.

Since the experimental observation revealed that the pyridinium moiety is crucial for hydrolysis, therefore, it became important to compare the catalytic activity of complexes with and without pyridine coordination with a transition metal. The coordination of 4C with Pd(H_2_O)_2_ yielded complex Int5, in which the pyridine moiety did not coordinate with the transition metal (Pd). The complex Int5 was 29.02 kcal mol^−1^ lower in energy than its reactants. Thiophene was coordinated with Pd through a η2 coordination. The Pd-C2 and Pd-C3 bond lengths were 2.13 Å and 2.14 Å. The water molecules were also coordinated to Pd, whereas the other water molecule was not bound to Pd, rather, it made a hydrogen bond with the coordinated water molecule (the Pd-H1 and Pd-H2 bond lengths were 3.20 Å and 3.47 Å). The coordination of another water molecule with complex Int5 resulted in the formation of complex Int7, which was about 4.9 kcal mol^−1^ more stable than complex Int7 (Figure 2). The coordinated water molecule interacted with palladium through the hydrogen of the water molecule. The Pd-H bond length in complex Int7 was 3.20 Å. Addition of water in complex Int7 was a kinetically demanding step with an activation barrier of 37.57 kcal mol^−1^. The activation barrier was significantly reduced, compared to that of the transition metal-free hydration. The geometries of the transition states were compared to find out the reasons for this difference in activation barriers. The transition state TS8 was late in nature, where the C-O and N-H bonds were significantly developed and the O-H bond was broken. The C-O bond length in TS8 was 1.55 Å compared to 1.74 Å in TS2. The hydration with Pd was thermodynamically favorable by 6.21 kcal mol^−1^, which was significantly larger than 2.37 kcal mol^−1^ for the metal-free reaction.

**Figure 3:**
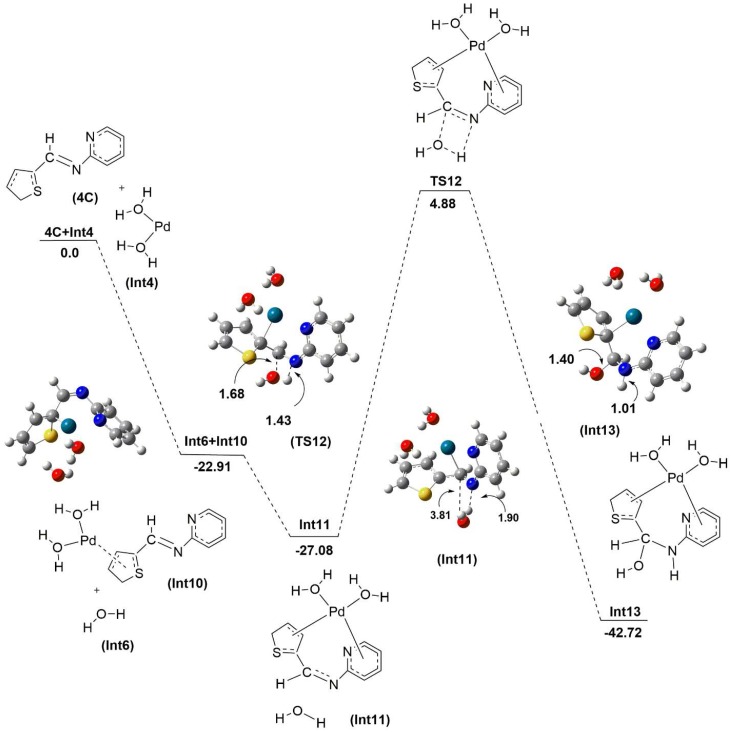
Hydrolysis pathway with palladium along with involvement of a pyridine moiety.

The coordination of compound 4C with Pd(H_2_O)_2_ could also generate complex Int10, where the pyridine moiety interacted with palladium. The complex formation energy of Int10 was −22.91 kcal mol^−1^. The palladium in complex Int10 showed coordination from an atom of thiophene and from the nitrogen of pyridine. The coordination of thiophene in complex Int10 was different than that of Int5 because the thiophene in 8 showed coordination from C2 and C3 (vide supra). Quite similar to complex Int5, one water molecule coordinated with the palladium, whereas the second water molecules showed hydrogen bonding interactions with the coordinated water molecule. The coordination of another water molecule resulted in structure Int11, which was about 4.17 kcal mol^−1^ lower in energy than Int10. The interaction energy of water in complex Int11 was quite comparable to that in complex Int7. The activation barrier for the crucial hydration step was 31.96 kcal mol^−1^, about 5.61 kcal mol^−1^ lower than the barrier without pyridine involvement. These observations were consistent with the experiment where pyridine involvement facilitated the hydrolysis. The C-O and N-H bond lengths in TS12 were 1.68 Å and 1.43 Å compared to 1.55 Å and 1.50 Å in TS8. While the O-H bond lengths were 0.98 Å and 1.01 Å, respectively, in TS12 and TS8. The bond length of N-H is a decisive factor in reducing the activation barrier. The reaction was, thermodynamically, highly favorable because the products were about 15.64 kcal mol^−1^ lower in energy than complex Int11 (see Figure 3). The high exothermicity of the reaction was probably a key factor for the low activation barrier in the crucial step. The activation barrier for hydration through pyridine involvement was 31.96 kcal mol^−1^, which was marginally accessible under the experimental reaction conditions. The activation barrier was slightly high, mainly due to the involvement of the four-membered ring. An alternative transition state with the involvement of an additional water molecule to form a six-membered transition state might further lower the activation barrier. Such lowering of the activation barrier with the involvement of the Brønsted acid species BH^+^ is well documented in the literature [23]. In summary, the theoretical calculations were strongly supportive of the experiment and provided an insight into the transition metal-catalyzed hydrolysis of imines.

## 3. Materials and Methods

### 3.1. General Information

The Buchi B-540 was used for determining the melting points of the synthesized products (Buchi, New Castle, DE, USA). ^1^H-NMR and ^13^C-NMR spectra were obtained in chloroform-d at 500/126, 400/100, MHz (Bruker, Billerica, MA, USA). The EI-MS spectra were recorded using the JMS-HX-110 spectrometer (JEOL, Peabody, MA, USA). TLC (Merck silica gel 60 PF_254_ cards) was used for monitoring the reaction. A UV lamp was used for visualization of the compounds (254–365 nm). The dry ethanol used in the experiments was dried by following a previously reported method [24].

### 3.2. General Synthetic Procedure of Schiff Bases

Aminopyridine (18.49 mmol, 1 eq) in ethanolic solution (100 mL) was reacted with bromo-substituted thiophene aldehydes (20.34 mmol, 1.1 eq) in the presence of glacial acetic acid (a few drops). For 6–8 h, the mixture was refluxed in a water bath at 80–85 °C. The completion of the reaction was monitored by TLC (chloroform:benzene 1:2). After cooling the mixture, the Schiff bases were purified by column chromatography and recrystallized using methanol [25].

### 3.3. General Procedure for Suzuki Coupling of Schiff Base with Aryl/het-Aryl Boronic Acids

The catalyst Pd(PPh_3_)_4_ (5 mol%) was added in the Schiff bases (0.712 mmol, 1 eq), under an inert nitrogen atmosphere. The reaction mixture was stirred for 30 min after addition of a 1,4-dioxane solvent (8 mL). Then, aryl/het-aryl boronic acids (0.783 mmol, 1.1 eq), K_3_PO_4_ (1.43 mmol, 2 eq) and H_2_O (2 mL) were added [26,27] and stirring of mixture was done for 20–25 h at 90 °C. The mixture was diluted with ethyl acetate at room temperature. The separated organic layer was dried with magnesium sulphate (MgSO_4_) and the solvent was removed under a vacuum. The crude product was purified by column chromatography using ethyl-acetate and *n*-hexane. For characterization of synthesized products, different spectroscopic techniques were used.

### 3.4. Characterization Data

*(E)-1-(3-bromothiophen-2-yl)-N-(4-methylpyridin-2-yl)methanimine* (**3a**): Brown solid, m.p. = 60 °C, (411 mg, 79%), ^1^H-NMR (500 MHz): δ 8.60 (s, 1H), 7.56 (d, *J* = 8.0, 2H), 7.43–7.37 (m, 1H), 6.51–6.48 (m, 2H), 2.29 (s, 3H); ^13^C-NMR (126 MHz): δ 153.0, 152.1, 149.7, 146.0, 130.1, 125.0, 123.0, 123.9, 115.9, 112.0, 20.7. EI/MS *m*/*z* (%): 282.0 [M + H]^+^, 283.7 [M + 2]; [M − CH_3_] = 265.3, [M − Br] = 201.4.

*(E)-1-(5-bromothiophen-2-yl)-N-(4-methylpyridin-2-yl)methanimine* (**3b**)*:* Off-white solid, m.p. = 60 °C, (390 mg, 75%) ^1^H-NMR (500 MHz): δ 8.62 (s, 1H), 7.62 (d, *J* = 9.0, 1H), 7.42-7.28 (m, 2H), 6.41–6.38 (m, 2H), 2.24 (s, 3H); ^13^C-NMR (126 MHz): δ 158.0, 154.0, 152.1, 149.0, 146.1, 133.0, 127.0, 125.0, 119.0, 115.0, 22.8. EI/MS *m*/*z* (%): 282.0 [M + H]^+^, 283.4 [M + 2]; [M − C_4_H_2_S, Br fragments] = 119.1, [M − Br] = 202.0.

*(E)-1-(3-bromothiophen-2-yl)-N-(6-methylpyridin-2-yl)methanimine* (**3c**)*:* Yellow solid, m.p. = 80 °C, (416 mg, 80%) ^1^H-NMR (500 MHz): δ 8.61 (s, 1H), 7.57–7.38 (m, 2H), 6.51 (d, *J* = 8.0, 1H), 6.43–6.35 (m, 2H), 2.38 (s, 3H); ^13^C-NMR (126 MHz): δ 161.0, 158.1, 152.6, 140.1, 126.1, 124.1, 123.7, 122.3, 114.6, 111.0, 25.1. EI/MS *m*/*z* (%): 282.5 [M + H]^+^, 283.0 [M + 2]; [M − Br] = 201.3.

*3-(4-chlorophenyl)thiophene-2-carbaldehyde* (**5a**): Yellow solid, m.p. = 90 °C, (114 mg, 72%). ^1^H-NMR (400 MHz): δ 9.21 (s, 1H), 7.51–7.46 (m, 3H), 7.42–7.39 (m, 3H); ^13^C-NMR (100 MHz, chloroform-d): δ 179.0, 150.1, 143.0, 139.0, 132.9, 131.9, 128.0, 127.1, 126.9, 126.0, 122.1. EI/MS *m*/*z* (%): 222.0 [M + H]^+^, 223.0 [M + 2]; [M − Cl] = 186.9.

*3-(3-chlorophenyl)thiophene-2-carbaldehyde* (**5b**)*:* Brown solid, m.p. = 190 °C, (73 mg, 46%). ^1^H-NMR (400 MHz) δ 9.61 (s, 1H), 7.55–7.53 (m, 1H), 7.44 (t, *J* = 1.8 Hz, 1H), 7.43–7.40 (m, 2H), 7.38(d, *J =* 4.0 Hz, 1H), 7.36-7.33 (m, 1H); ^13^C-NMR (100 MHz): δ 180.0, 151.1, 142.0, 140.1, 135.1, 132.1, 128.0, 127.1, 126.9, 125.0, 122.9. EI/MS m/z (%): 222.0 [M + H]^+^, 223.0 [M + 2]; [M − CHO] = 193.2.

*3-(3,4-dichlorophenyl)thiophene-2-carbaldehyde* (**5c**)*:* Brown solid, m.p. = 120 °C, (61 mg, 30%). ^1^H-NMR (400 MHz) δ 9.65 (s, 1H), 7.70–7.61 (m, 2H), 7.52 (dd, *J* = 10.4, 4.2 Hz, 1H), 7.48–7.41 (m, 2H); ^13^C-NMR (100 MHz): δ 181.0, 150.1, 143.0, 140.4, 135.6, 132.8, 130.0, 129.1, 128.0, 126.0, 124.1. EI/MS *m*/*z* (%): 257.0 [M + H]^+^, 258.0 [M + 2]; 259.0 [M + 4]; [M − Cl] = 220.4. [M − 2Cl] = 186.2.

*3-(3-chloro-4-fluorophenyl)thiophene-2-carbaldehyde* (**5d**): Yellow solid, m.p. = 110 °C, (77 mg, 45%). ^1^H-NMR (400 MHz) δ 9.72 (s, 1H), 7.82 (d, *J =* 7.0, 2H), 7.27 (d, *J* = 6.4 Hz, 1H), 7.10 (m, 2H); ^13^C-NMR (100 MHz): δ 180.1, 159.0, 151.3, 143.0, 141.2, 134.6, 130.7, 129.0, 125.0, 123.7, 115.1. EI/MS *m*/*z* (%): 240.0 [M + H]^+^, 241.0 [M + 2]; [M − Cl] = 205.4. [M − Cl, F] = 186.2.

*3-(4-(methylthio)phenyl)thiophene-2-carbaldehyde* (**5e**)*:* Brown solid, m.p. = 160 °C, (142 mg, 85%). ^1^H-NMR (400 MHz) δ 9.54 (s, 1H), 7.51–7.50 (m, 2H), 7.49–7.48 (m, 2H), 7.34-7.32 (m, 1H), 7.30-7.31 (m, 1H), 2.52 (s, 3H); ^13^C-NMR (100 MHz): δ 181.1, 154.0, 144.0, 143.2, 138.6, 134.0, 128.0, 127.0, 126.9, 126.0, 124.0, 14.1. EI/MS *m*/*z* (%): 235.1 [M + H]^+^, [M − SCH_3_] = 186.9, [M − SCH_3_, CHO] = 158.2.

*3-(3-acetylphenyl)thiophene-2-carbaldehyde* (**5f**)*:* Brown solid, m.p. = 110 °C, (104 mg, 65%). ^1^H-NMR (400 MHz) δ 9.44 (s, 1H), 8.22 (t, *J =* 1.8 Hz, 2H), 7.98 (d, *J =* 1.0 Hz, 1H), 7.86 (d, *J =* 4.0 Hz, 1H), 7.59 (t, *J =* 7.7 Hz, 2H), 2.62 (s, 3H); ^13^C-NMR (100 MHz): δ 198.1, 183.1, 153.0, 142.0, 140.1, 139.6, 137.0, 130.0, 128.0, 127.0, 125.0, 124.8, 25.1. EI/MS *m*/*z* (%): 231.1 [M + H]^+^, [M − COCH_3_] = 187.1, [M − COCH_3_, CHO] = 158.1.

*3-(3,5-dimethylphenyl)thiophene-2-carbaldehyde* (**5g**)*:* Brown solid, m.p. = 130 °C, (60 mg, 38%). ^1^H-NMR (400 MHz) δ 9.34 (s, 1H), 7.68–7.63 (m, 2H), 7.52 (d, *J* = 7.3 Hz, 1H), 7.45 (d, *J* = 6.2 Hz, 2H), 2.01 (s, 6H); ^13^C-NMR (100 MHz, chloroform-d): δ 184.1, 153.2, 145.1, 141.1, 140.1, 138.1, 134.1, 130.5, 126.1, 125.1, 122.1, 22.9, 20.9. EI/MS m/z (%): 217.3 [M + H]^+^, [M − CH_3_] = 202.1, [M − 2CH_3_] = 187.1.

*3-(4-methoxyphenyl)thiophene-2-carbaldehyde* (**5h**): Brown solid, m.p. = 140 °C, (65 mg, 42%). ^1^H-NMR (400 MHz) δ 9.99 (s, 1H), 7.51–7.48 (m, 3H), 6.98 (d, *J =* 7.0 Hz, 2H), 6.95 (d, *J =* 2.1 Hz, 1H), 3.85 (s, 3H); ^13^C-NMR (100 MHz): δ 181.1, 161.1, 153.4, 141.3, 140.0, 129.0, 128.0, 127.2, 122.1, 113.1, 112.7, 56.1. EI/MS *m*/*z* (%): 219.6 [M + H]^+^, [M − OCH_3_] = 186.9, [M − CHO, OCH_3_] = 158.1.

*methyl 4-(2-formylthiophen-3-yl)benzoate* (**5i**): Brown solid, m.p. = 105 °C, (70 mg, 40%). ^1^H-NMR (400 MHz _3_) δ 9.79 (s, 1H), 8.13 (d, *J* = 8.3 Hz, 2H), 7.95 (d, *J* = 6.0 Hz, 1H), 7.69 (d, *J* = 8.3 Hz, 2H), 6.88 (d, *J* = 8.7 Hz, 1H), 3.89 (s, 3H); ^13^C-NMR (100 MHz): δ 183.7, 167.1, 154.1, 142.1, 140.1, 139.0, 131.1, 129.0, 128.1, 127.1, 125.0, 122.1, 52.0. EI/MS *m*/*z* (%): 247.6 [M + H]^+^, [M − COOCH_3_] = 187.4, [M − CHO, COOCH_3_] = 158.1.

*5-bromo-[2,3′-bithiophene]-2′-carbaldehyde* (**5j**): Brown solid, m.p. = 140 °C, (76 mg, 39%). ^1^H-NMR (400 MHz) δ 9.80 (s, 1H), 7.66 (dd, *J* = 10.1, 4.2 Hz, 1H), 7.54 (t, *J* = 7.3 Hz, 1H), 7.47-7.45 (m, 2H), ^13^C-NMR (100 MHz): δ 181.7, 154.1, 142.6, 141.1, 139.0, 133.1, 129.9, 128.1, 111.0, EI/MS *m*/*z* (%): 272.0 [M + H]^+^, 273.0 [M + 2], [M − Br] = 193.1, [M − Br, CHO] = 165.0.

*3-(2,3-dichlorophenyl)thiophene-2-carbaldehyde* (**5k**): Brown solid, m.p. = 102 °C, (82 mg, 45%). ^1^H-NMR (400 MHz) δ 9.79 (s, 1H), 7.70–7.63 (m, 2H), 7.58–7.52 (m, 1H), 7.47–7.45 (m, 2H),^13^C-NMR (100 MHz): δ 182.1, 150.5, 144.1, 141.4, 134.6, 131.8, 130.0, 129.1, 128.0, 126.1, 123.0. EI/MS *m*/*z* (%): 257.0 [M + H]^+^, 258.0 [M + 2]; 259.0 [M + 4]; [M − 2Cl] = 186.2.

*5-(4-chlorophenyl)thiophene-2-carbaldehyde* (**6a**): White solid, m.p. = 110 °C, (63 mg, 40%). ^1^H-NMR (400 MHz) δ 9.31 (s, 1H), 7.76 (d, *J* = 8.1 Hz, 3H), 7.45 (d, *J* = 6.1 Hz, 3H); ^13^C-NMR (100 MHz): δ 180.0, 151.2, 149.0, 142.0, 136.0, 132.9, 128.0, 127.1, 126.9, 126.0, 122.1. EI/MS *m*/*z* (%): 222.0 [M + H]^+^, 223.0 [M + 2]; [M − Cl] = 187.2.

*5-(3-chlorophenyl)thiophene-2-carbaldehyde* (**6b**): Brown liquid, (76 mg, 48%). ^1^H-NMR (400 MHz) δ 9.21 (s, 1H), 7.72–7.70 (m, 3H), 7.54 (d, *J* = 5.8 Hz, 1H), 7.23–7.20 (m, 2H): ^13^C-NMR (100 MHz): δ 181.0, 152.1, 143.0, 141.1, 139.1, 135.2, 129.0, 128.1, 125.3, 124.0, 121.9. EI/MS *m*/*z* (%): 222.0 [M + H]^+^, 223.0 [M + 2]; [M − CHO, Cl] = 161.0.

*5-(3,4-dichlorophenyl)thiophene-2-carbaldehyde* (**6c**): White solid, m.p. = 110 °C, (73 mg, 40%). ^1^H-NMR (400 MHz) δ 9.85 (s, 1H), 7.80 (d, *J* = 8.4 Hz, 2H), 7.42 (dd, *J* = 8.4, 2.2 Hz, 1H), 7.32-7.31 (m, 2H); ^13^C-NMR (100 MHz): δ 182.0, 154.1, 146.0, 144.4, 133.0, 132.0, 130.0, 129.6, 127.0, 126.1, 12431. EI/MS m/z (%): 257.0 [M + H]^+^, 258.0 [M + 2]; 259.0 [M + 4]; [M − 2Cl] = 186.0.

*5-(3-chloro-4-fluorophenyl)thiophene-2-carbaldehyde* (**6d**): Brown solid, m.p. = 80 °C, (60 mg, 35%). ^1^H-NMR (400 MHz) δ 9.78 (s, 1H), 7.66–7.63 (m, 1H), 7.58–7.54 (m, 1H), 7.49–7.44 (m, 1H), 6.94–6.92 (m, 1H), 6.87 (t, *J* = 8.0 Hz, 1H); ^13^C-NMR (100 MHz): δ 181.1, 153.0, 151.0, 142.0, 141.0, 133.5, 131.4, 128.0, 126.0, 124.0, 114.1. EI/MS *m*/*z* (%): 240.0 [M + H]^+^, 241.5 [M + 2]; [M − Cl, CHO] = 176.3.

*5-(4-(methylthio)phenyl)thiophene-2-carbaldehyde* (**6e**): White solid, m.p. = 180 °C, (73 mg, 44%). ^1^H-NMR (400 MHz) δ 9.64 (s, 1H), 7.76 (d, *J* = 6.8 Hz, 2H), 7.69–7.68 (m, 3H), 7.53–7.52 (m, 1H), 2.62 (s, 3H); ^13^C-NMR (100 MHz, chloroform-d): δ 183.1, 155.0, 146.0, 144.0, 139.6, 136.0, 129.0, 127.6, 126.0, 125.0, 122.0, 15.1. EI/MS *m*/*z* (%): 235.1 [M + H]^+^, [M − SCH_3_] = 186.7, [M − SCH_3_, CHO] = 158.0.

*5-(3-acetylphenyl)thiophene-2-carbaldehyde* (**6f**): Brown solid, m.p. = 80 °C, (98 mg, 60%). ^1^H-NMR (400 MHz) δ 9.50 (s, 1H), 8.19 (m, 1H), 7.96 (d, *J* = 8.0 Hz, 1H), 7.81 (d, *J* = 8.0 Hz, 1H, 7.58–7.47 (m, 2H), 7.33-7.29 (m, 1H), 2.67 (s, 3H); ^13^C-NMR (100 MHz): δ 197.0, 185.1, 154.0, 144.0, 143.1, 139.0, 136.2, 132.0, 128.4, 126.0, 125.5, 124.0, 24.0. EI/MS *m*/*z* (%): 231.3 [M + H]^+^, [M − CHO] = 201.4, [M − COCH_3_, CHO] = 158.3.

*5-(3,5-dimethylphenyl)thiophene-2-carbaldehyde* (**6g**): Brown solid, m.p. = 130 °C, (62 mg, 40%). ^1^H-NMR (400 MHz) δ 9.56 (s, 1H), 7.69–7.64 (m, 2H), 7.55–7.52 (m, 2H), 7.47–7.45 (m, 1H), 2.65 (s, 6H); ^13^C-NMR (100 MHz,): δ 183.1, 154.2, 146.1, 143.0, 141.1, 139.1, 135.1, 131.0, 125.1, 124.1, 122.0, 21.8, 20.5. EI/MS *m*/*z* (%): 217.3 [M + H]^+^, [M − CH_3_] = 202.1, [M − CHO] = 187.2.

*5-(4-methoxyphenyl)thiophene-2-carbaldehyde* (**6h**): White solid, m.p. = 150 °C, (92 mg, 59%). ^1^H-NMR (400 MHz) δ 9.89 (s, 1H), 7.69–7.64 (m, 2H), 7.56–7.52 (m 2H), 7.47–7.44 (m, 2H), 2.53 (s, 3H); ^13^C-NMR (100 MHz): δ 182.1, 162.1, 154.0, 142.0, 141.0, 130.0, 129.0, 128.2, 124.1, 115.1, 114.7, 54.1. EI/MS *m*/*z* (%): 219.4 [M + H]^+^, [M − CHO] = 189.1, [M − OCH_3_] = 186.7.

*5′-bromo-[2,2′-bithiophene]-5-carbaldehyde* (**6i**): Brown solid, m.p. = 140 °C, (68 mg, 45%). ^1^H-NMR (400 MHz) δ 9.90 (s, 1H), 7.84 (d, *J* = 8.1, 4.1 Hz, 2H), 7.54 (m, *2*H); ^13^C-NMR (100 MHz): δ 184.0, 153.0, 143.0, 141.7, 139.5, 134.0, 130.1, 127.1, 112.0, EI/MS *m*/*z* (%): 272.2 [M + H]^+^, 273.3 [M + 2], [M − CHO] = 243.0. [M − Br] = 193.5.

### 3.5. Computational Methods

For the density functional theory (DFT) studies, the Gaussian 09 software was used [28]. For visualization of the results, GaussView 5.0 was used [29]. The reactants, transition states, intermediates, and products were optimized by using ωB97XD along with the split basis set approach. LANL2DZ was implemented for Pd and 6-31G(d) was for all other atoms. The reactants and products were confirmed with no imaginary value of frequency, whereas for the transition states one imaginary frequency was observed.

## 4. Conclusions

Coupling of a Schiff base (**3a**, **3b**, **3c**) with aryl/het-aryl boronic acids under Suzuki coupling reaction conditions yielded products with hydrolysis of the imine linkage (**5a**–**5k**, **6a**–**6h**) in good to moderate yields. To gain mechanistic insight into the transition metal-catalyzed hydrolysis of the compounds, theoretical calculations were conducted, which strongly supported the experiment and provided insight into the transition metal-catalyzed hydrolysis of imines. DFT calculations proved that the presence of the pyridine moiety was crucial for the hydrolysis of imine. The activation barrier for the crucial hydration step, in case of pyridine, was 31.96 kcal mol^−1^, which was about 5.61 kcal mol^−1^ lower than the activation barrier without pyridine involvement.

## Figures and Tables

**Table 1:**
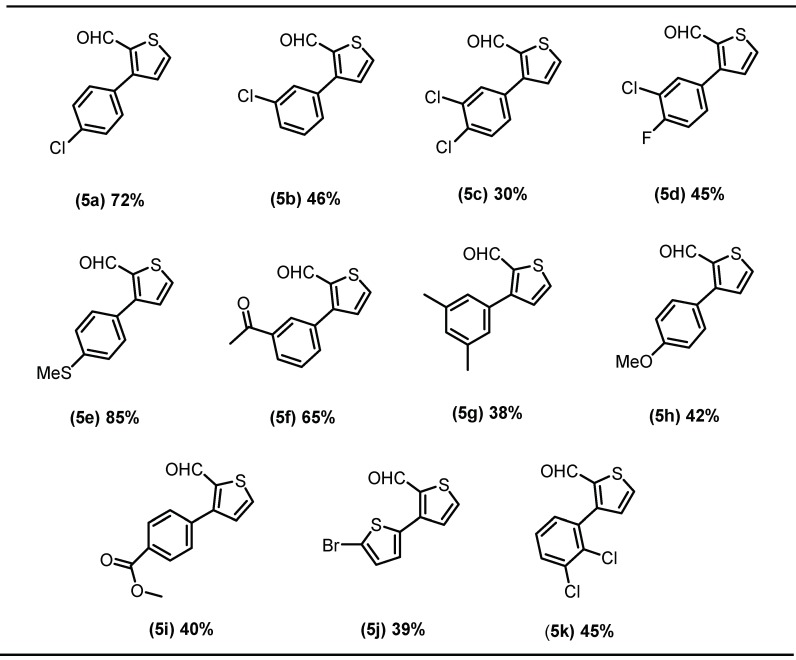
Hydrolytic products obtained after Suzuki coupling of (E)-1-(3-bromothiophen-2-yl)-N-(4-methylpyridin-2-yl)methanimine (**3a**) with Aryl/het-aryl boronic acids.

**Table 2:**
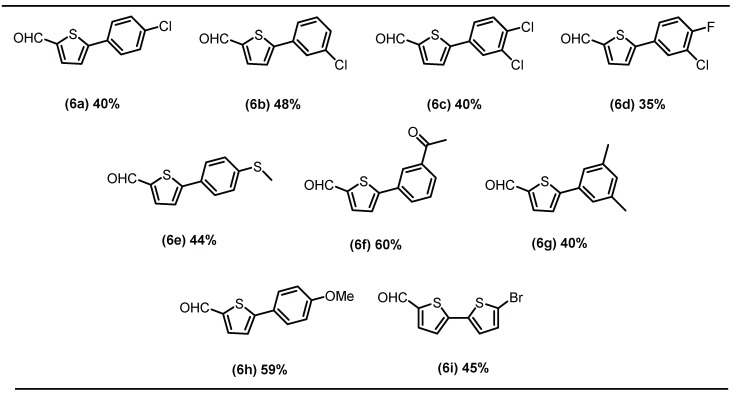
Hydrolytic products obtained after Suzuki coupling of (E)-1-(5-bromothiophen-2-yl)-N-(4-methylpyridin-2-yl)methanimine (**3b**) with Aryl/het-aryl boronic acids.
